# Therapeutic application of nanotechnology in cardiovascular and pulmonary regeneration

**DOI:** 10.5936/csbj.201304005

**Published:** 2013-09-21

**Authors:** Young Wook Chun, Spencer W Crowder, Steven C Mehl, Xintong Wang, Hojae Bae, Hak-Joon Sung

**Affiliations:** a Department of Biomedical Engineering, Vanderbilt University, Nashville, TN, USA; b Department of Maxillofacial Biomedical Engineering, Kyung Hee University, Seoul, S.Korea

## Abstract

Recently, a wide range of nanotechnologies has been approached for material modification by realizing the fact that the extracellular matrix (ECM) consists of nanoscale components and exhibits nanoscale architectures. Moreover, cell-cell and cell- ECM interactions actively occur on the nanoscale and ultimately play large roles in determining cell fate in tissue engineering. Nanomaterials have provided the potential to preferentially control the behavior and differentiation of cells. The present paper reviews the need for nanotechnology in regenerative medicine and the role of nanotechnology in repairing, restoring, and regenerating damaged body parts, such as blood vessels, lungs, and the heart.

## Introduction

Regenerative medicine holds great promise for restoring the normal, healthy functions of human tissues after damage. Its potential to treat a broad range of degenerative and ischemic diseases in tissues or organs has been improved with significant progress in understanding biological mechanisms. Based on current technologies, growing tissues and organs in the laboratory becomes a reality in regenerative medicine [1]. Tissue engineering is a central tenet of regenerative medicine. The purpose of tissue engineering is not only to repair damaged organs and tissues, but also to grow healthy ones to replace their damaged counterparts in patients [2].

Currently, engineered biomaterial scaffolds with biological functionalization through cell seeding have been widely used to regenerate healthy tissues for replacement. Instead of simply introducing healthy cells into a diseased region, cells are actually seeded onto biomaterial scaffolds before transplantation [3]. These biomaterials serve as instructive templates for cell growth and tissue architecture so that functional tissue can eventually be formed. Therefore, this ultimate outcome can address the urgent issue related to available tissue and organs for patients who are awaiting life-saving transplantation. Selection of synthetics or natural materials as well as appropriate choice of cell type provides numerous options to develop various types of tissues and organs. Studies have begun to shed light on the significance of nanoscale interactions between cells and scaffolds [1, 4]. Recently, a wide range of nanotechnologies for material modification has been approached by realizing the fact that the extracellular matrix (ECM) consists of nanoscale components and exhibits nanoscale architectures. Moreover, cell-cell and cell-ECM interactions actively occur on the nanoscale and ultimately play large roles in determining cell fate [5]. These cell-ECM interactions are based on topography, mechanical properties (e.g. matrix stiffness, viscosity and elasticity), concentration gradients of arrested growth factors, and ECM molecules. For example, the importance of cell-ECM interactions was demonstrated by Ott and co-workers [6]. The ECM is composed of an intricate interweaving of protein fibers such as fibrillar collagen and elastins which range from 10 to hundreds of nanometers. This mesh is coated with nanoscale adhesion proteins like laminin and fibronection which allow for cell adhesion and cell-matrix interaction. In this study, rat hearts were decellularized by the perfusion of detergents, resulting in preservation of the fundamental ECM structure. The researchers observed that collagens I and III, laminin, and fibronectin remained within the decellularized heart, proving that the integrity of the ECM was kept intact. When the decellularized heart was reseeded with cardiac and endothelial cells, the cells migrated and self-organized into their natural physiological location. By day 8, the cells were even able to generate a pump function under both physiological loading and electrical stimulation. Similar studies have been conducted for liver [7], bone [8], lung [9], and arteries [10]. These works show that for each organ system there is a specific environment (e.g., tissue architecture) that helps direct cell fate.

Nanomaterials have provided the potential to preferentially control the behavior and differentiation of cells by controlling nanoscale properties [4]. With this foundation, the current review is focused on the needs of nanotechnology in developing tissue engineered scaffolds and the role of nanotechnology in improving tissue growth and function or inhibiting abnormal cell proliferation for major organs found in both the pulmonary and cardiovascular systems.

## 1. The need of nanotechnology for regenerative medicine

**Figure 1:**
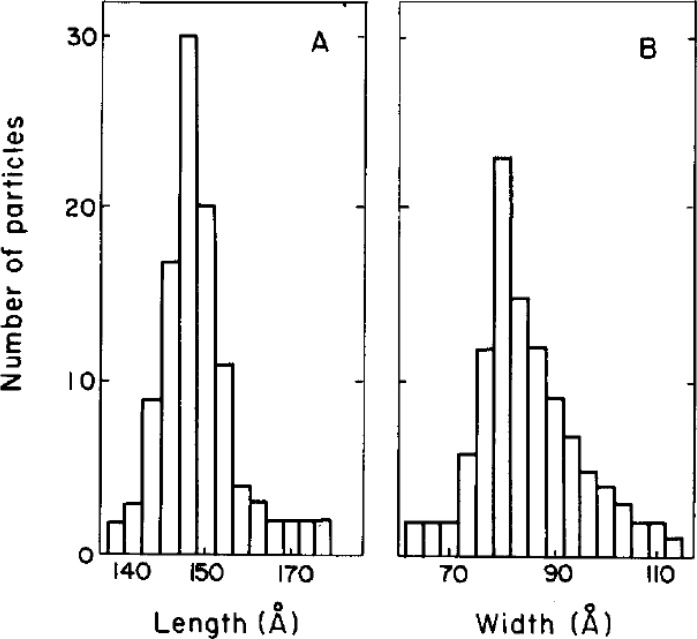
Koteliansky *et. al*. measured length (**A**) and width (**B**) of fibronectins using electron microscopy.

Nanoscale materials and therapeutics have been shown to play significant roles in tissue engineering applications since cells respond to nanoscale stimuli in spatial parameters [1, 4, 11]. The goal of tissue engineering is to build a natural tissue or organ for replacement of the damaged body part. This task could be done more effectively, if the spatiotemporal profile in expression of key molecules (e.g., proteins and polysaccharides) regulating cell behavior can be precisely controlled by means of nanotechnology. Although the size of most human cells is in the microscale range (10 to 100 µm), biological molecules that play crucial roles in virtually all mechanisms of cell function (i.e. adhesion, differentiation and proliferation) are much smaller in size. Specifically, the active sites of adhesion proteins, such as those associated with ligand-receptor interactions, cell-cell junctions, and cell-ECM binding, are on the order of nanometers. For example, the size of fibronectin, a critical protein in cell adhesion and proliferation, is approximately 20nm and 10nm in length and width, respectively (Figure 1), and its active site is approximated to be much smaller [12]. This means that nanoscale engineering approaches can directly influence the response of key molecules in cells and eventually change overall cellular behavior, which will ultimately improve tissue-level functions.

It is truly challenging to engineer ECM proteins and/or nanomaterial surfaces to function similar to the natural ECM proteins in tissues and organs. However, there have been enormous endeavors towards this goal through nanotexturing, nanopatterning and nanomaterials, although these techniques still need to be further developed [11, 13–15].

ECM structure is also important to mimic because it generates the level of integrity required for structural support to resident cells, as well as regulating a deposition pattern of growth factors [16]. For example, collagens are the most abundant proteins in ECM and are present as a form of fibrillar proteins. In normal tissues, the anchoring fibrils represent tissue structures with nanoscale diameters, but in pathological tissue, collagen fibril breakage and abnormal aggregation result in disorganized ECM with altered fibril diameters. Therefore, well-organized ECM represents the healthy environment, preventing cells from being exposed to pathological environments. In order to achieve a similar organization via engineering techniques, electrospinning can be employed for generating synthetic ECM “fibrils” that are structurally similar to those found in normal physiology [17, 18]. Moreover, the resulting fibers can be tailored on the nanoscale size and functionalized to deliver biological components.

Cells also encounter and interact with many topographical features which can range from folded proteins to banded collagen [19]. This important interaction can be utilized through surface modifications of biomaterial scaffolds. Surface modification can be done by nanopatterning different geometries onto the surface of a given scaffold [20, 21]. These geometries include nanogrooves, nanoposts, and nanopit arrays [19]. The topography of different substrates influences a wide range of cellular functions, including morphological changes, differentiation, and adhesion. An example of this can be seen when epithelial cells are seeded on surfaces of varying topographies. Epithelial cells elongate and align along grooves and ridges as small as 70 nm, but the cells are mostly round on smooth substrates [22].

Nanoparticles are also an exciting subject in nanotechnology that has been investigated in the field of regenerative medicine. These particles are usually loaded with therapeutics and tagged with appropriate antibodies. These antibodies are specific to ligands in the diseased tissue so the nanoparticles circulate in the body for a long period and bind to the desired tissue. A current method of treatment is the intravenous injection of therapeutic agents [23–27]. The major limitation of this treatment method is the lack of retention in the desired target tissue partially due to inefficient targeting. Another treatment involves transplantation of biomaterials, thereby enabling sustained release of biomolecules, but this involves invasive surgery [28–30]. Targeted nanoparticles are more favorable than either of these current treatments because of their improved retention and minimally invasive administration. There are two primary obstacles that must be overcome in order to be a successful targeting vehicle. The first obstacle is the delivery vehicle must be transferred across the vascular endothelium, which is difficult under normal circumstances. A phenomenon known as enhanced permeation and retention (EPR) is utilized to ensure the successful transport of nanoparticles across the endothelium. This process is primarily seen in tumor vasculature that is characterized by the increased endothelial permeability. The second obstacle that must be addressed is a molecular target must be identified at the desired site of drug action so that a much higher concentration of drug can be presented at the site compared to its systemic concentration.

The ECM of each organ in the body differs in their composition and spatial organizations [4]. The ECM maintains specific tissue morphologies and provides specific instructive cues that are key for operating each organ. Therefore, design considerations for scaffolds should vary accordingly for each desired organ. The biochemical, mechanical and electrical functions of the heart represent the importance of this specificity [31]. The three-dimensional ECM network of heart is made by intricate, micro and nanoscale interweaving of elastin and collagen. In this unique environment, cardiomyocytes form elongated and aligned cell bundles as they are forced to couple mechanically with each other and to communicate electrically through gap junctions. This multi-bundled elongated, connected structure is essential for creating the unique electrical and mechanical properties of the heart. A specific ECM is also defined for the pulmonary system to ensure appropriate diffusion of both carbon dioxide and oxygen across the alveolar cell wall. This is essential to enable the systemic distribution of oxygen. Many of the current therapeutic applications for both the pulmonary and cardiovascular system are based on an understanding of nanotechnology. A better understanding of these two systems on the nanoscale can lead to novel treatments that will advance regenerative medicine. Therefore, the following sections will discuss the details of nanotechnology approaches for recapitulating tissue- and organ-level functions in order to produce significant advances in the field of tissue engineering and regenerative medicine.

## 2. Blood vessel

Currently, there are a few conventional strategies used to enhance vascularization in tissue-engineered constructs. A favorable way to vascularize engineered tissues is to activate the natural angiogenic potential of the body [32]; however, the primary disadvantage of this approach is that it takes too long for whole implants to become properly perfused [33–37]. Instead, nano-structured or nano-surface modified vascular scaffolds can be employed to greatly influence cell alignment, adhesion and differentiation to promote more efficient and complete vascularization. Xu *et. al*. observed the behavior of smooth muscle cells (SMCs) on a biodegradable poly(L-lactide-*co*-*ɛ*-caprolactone) (75:25) scaffold fabricated to aligned nanofibrous structures by electrospinning [35]. When compared to identical microscale polymer scaffolds, the electrospun fibers with an aligned nonotopography showed improved SMC adhesion, proliferation and *in vivo*-like cell orientation and migration along the aligned nanofibers [35]. Tissue engineered vascular grafts have a number of requirements that must be met, including appropriate mechanical strength, prevention of surface thrombosis, and highly organized structures that combine with ECM proteins, such as collagen and elastin. For mechanical strength and highly organized structures, nanotechnology can be used as a means to create patterning on the nanoscale. One recent study developed tubular collagen scaffolds with nanopatterns (TNs) to mimic the native vasculature [37]. TNs were produced both inside and outside of the tubes, and endothelial cells were seeded on the luminal side while smooth muscle cells were seeded on the outside of the tubes. Following co-culturing in double-sided nanopatterned tubes, the phenotypes of both endothelial cell and smooth muscle cells were respectively identical to their healthy ones. The authors explained that TNs have potential for vascular tissue engineering since the TNs enhanced tensile strength while improving cell retention in the lumen under blood flow. Additionally, both cell types retained their proliferative capacity. Accordingly, another study showed the importance of strengthening the interaction with collagens on the nanoscale to increase mechanical property of scaffolds as well as to mimic ECM structure [37].

One of the largest causes of morbidity and mortality worldwide is ischemic tissue diseases. Therapeutic angiogenesis has emerged as a potential treatment plan because it enhances microvascular perfusion in ischemic tissue by delivering pro-angiogenic molecules. This treatment can be optimized through a better understanding of the nanoscale regulation of the process that involves both delivering growth factors and exerting complex signaling cascades. One of the most potent angiogenic factors that have been recognized is vascular endothelial growth factor (VEGF). Therefore, many pro-angiogenic treatments have attempted to utilize VEGF to enhance microvasculature in ischemic tissue. A primary obstacle of the treatments with VEGF is inadequate retention of the protein at the desired site of action. Webber et al., have developed a supramolecular nanostructure that mimics VEGF by reacting with VEGF receptors on the cell membrane [38]. This nanomaterial is a liquid that forms a matrix of loosely tangled nanofibers when it is injected into a patient. The nanofiber is covered in microscopic protuberances that mimic the physiological actions of VEGF. These protuberances were able to induce VEGF receptor phosphorylation and promote endothelial cells to exhibit proangiogenic behavior. When this VEGF-mimetic was investigated in a mouse hind-limb ischemia model, the nanostructure increased tissue perfusion, functional recovery, limb salvage, and treadmill endurance compared to controls. Unfortunately, this nanotechnology still does not address the main obstacle of poor retention [38]. However, this approach showed an important utility of nanotechnology for blood vessel regeneration.

## 3. Lung

**Figure 2:**
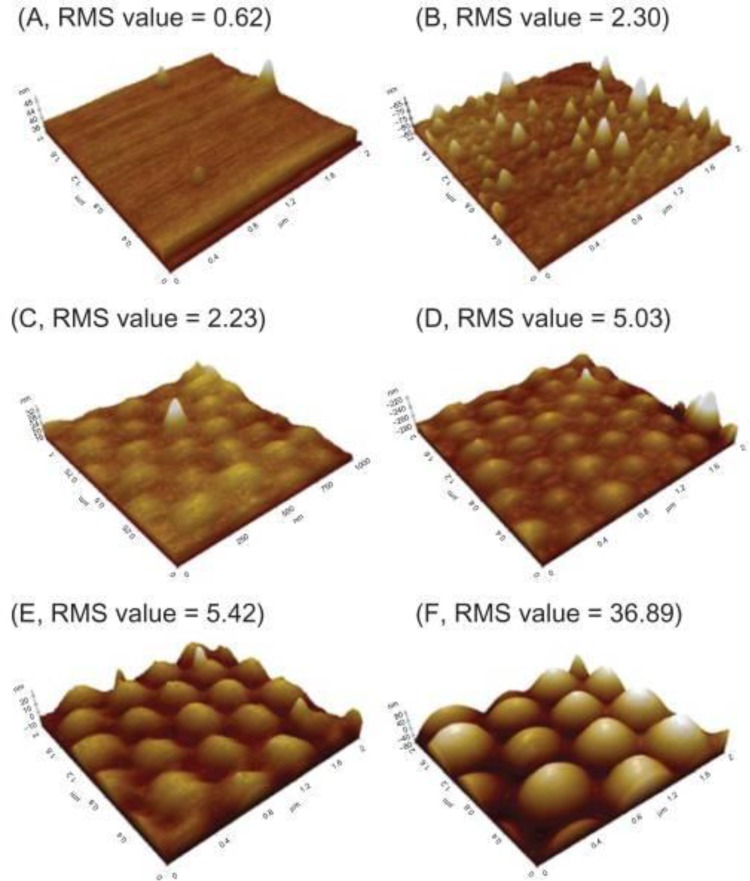
Zhang *et. al*. generated a wide range of nanoroughness on PLGA surfaces formed by polystyrene beads. RMS values of A, B, C, D, E and F showed 0.62, 2.30, 2.33, 5.03, 5.42 and 36.89, respectively.

Alterations to surface nanotopography have been employed to stimulate a wide range of cell functions. Nanopillars and nanolines [34] have been shown to effectively modify the dynamics of cell spreading in cancerous fibroblasts. Also the appropriate choice of nanoroughness on nanospherical surfaces [36] has been shown to adversely affect adhesion and proliferation of lung carcinoma cell lines. Both of these nanotechnologies have suggested that cancer cells respond to nanoscale changes in surface topography at the polymer surface. Zhang *et al*. have created a wide array of poly(lactic acid-*co*-glycolic acid) (PLGA) nanorough surfaces by using 190, 300, 400 and 530 nm polystyrene nanobeads through PDMS template molds (Figure 2) [36]. The nanoroughness of the surfaces was determined by the value of root mean square (RMS) of 2.23, 5.03, 5.42, and 36.90 nm. The authors seeded lung carcinoma cells on these surfaces and observed the subsequent cell behavior after three days. PLGA surfaces with an RMS value of 0.62 nm had the lowest cell density. The contact angle data on each PLGA surfaces exhibited the interrelationships between nanotopography and cell adhesion. The contact angle value on 0.62 nm rough surface was the lowest at approximately 100 degrees while control (glass) indicated the highest at approximately 60 degrees [36]. Considering the PLGA surface features are on the nanoscale, specific surface nanoroughness may inhibit adhesion of lung cancer cells when implanted. This result suggests a previously unknown biomaterial cue to inhibit lung cancer spreading.

Nanoparticles (NPs) are another nanotechnology with potential for interventional lung cancer treatment. NP research is currently one of the most intensive scientific interests due to a wide range of potential applications in the field of regenerative medicine. NPs can effectively carry drugs or small molecules in order to modulate functions at the cell- and tissue-level, exemplifying how nanotechnology can impact physiological function on a much larger scale. One recent study investigated the effects of magnetic NPs loaded with doxorubicin on lung cancer cell function [39]. The authors first synthesized chemically modified superparamagnetic iron oxide NPs using N-isopropylacrylamide and methacrylic acid (pNIPAAm-MAA) through covalent bonding, and then loaded the NPs with doxorubicin for delivery. Based on results from X-ray refraction desorption (XRD), the superparamagnetic iron oxide NPs ranged from 20-75nm. Following graft polymerization, the NPs increased in size to 60-100nm; also the dispersion of particles was greatly improved. They found that the pNIPAAm-MAA-grafted NPs demonstrated little cytotoxicity to lung cancer cells. Moreover, the temperature-dependent property of NIPAAm-MAA allowed grafted NPs to preserve the polymer benefits. Thus, doxorubicin-loaded NPs showed better drug release efficiency compared to doxorubicin alone in 37°C. The authors concluded that doxorubicin-loaded NPs could be potent inhibitors of lung cancer cells, but further studies are needed to confirm how healthy and cancerous cells respond to these NPs. These studies suggest that nanotechnology may contribute to the control of cancer cell growth through nanoscale manipulation.

## 4. Heart

**Figure 3:**
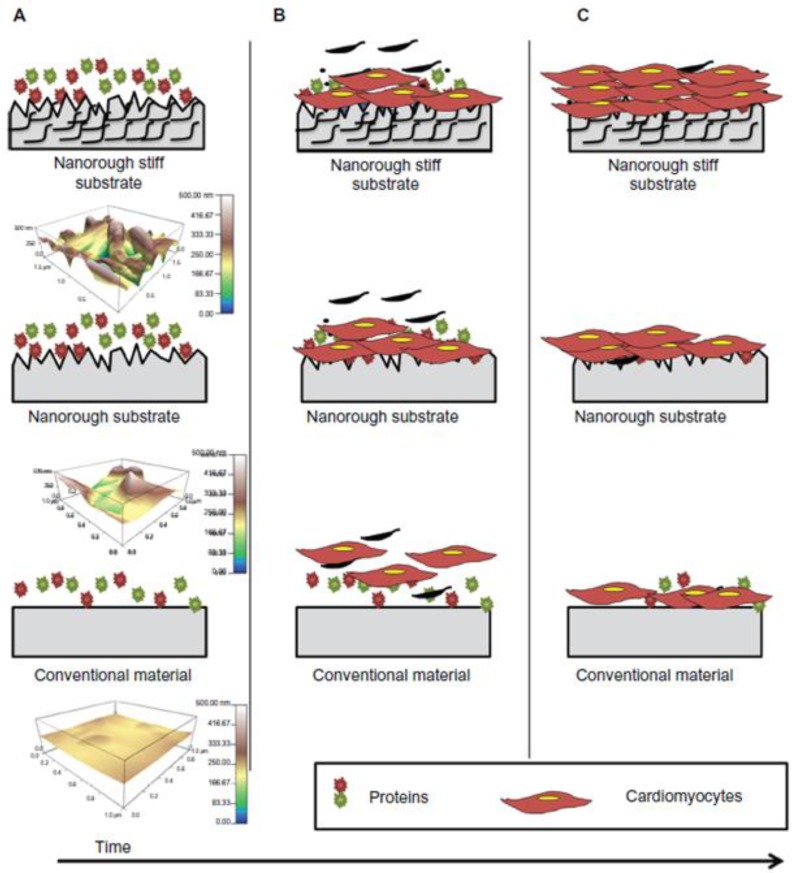
Suggested schematics of nanoroughness effects by Stout *et. al*. (**A**) Shows the adsorption of ECM proteins immediately when substrates implanted or soaked in media. (**B**) Indiactes the cardiomyocytes adhered to the substrates and begin to grow. (**C**) Due to mimicking native myocardium ECM in surface features, more cardiomyocytes on nanorough stiff substrates were adhered and grown than on conventional and plain nanorough substrates.

Nanoscale architecture plays a large role in manipulating protein adsorption, cell attachment, and cell functions [5]. When compared to plain surfaces, nanofibrous scaffolds have 2.6-3.9 times more protein adsorption, including ECM proteins and adhesion molecules [5]. Nanomaterials have also been shown to improve cardiomyocyte functions. A recent study reported that the addition of carbon nanofibers (CNFs) in PLGA scaffolds promoted cardiomyocyte growth and increased both the electrical conductivity and tensile strength of the scaffold as compared to conventional polymer substrates [40]. These results suggest that the CNFs help provide properties similar to those of natural cardiac tissue. The authors suggested that the reason for enhanced cardiomyocyte growth upon the addition of CNFs may be due to increased wettability of the surface, which results in improved adsorption of ECM proteins such as fibronectin and vitronectin, on the surface of scaffolds (Figure 3) [15]. Carbon nanotubes (CNTs) have been at the forefront of nanotechnology due to their unique electrical, mechanical, and thermal properties [4, 41–43]. CNTs have nanostructures with cylindrical shape and diameter ranging from 1 to 100 nm. CNTs can be functionalized by attaching chemical compounds or drugs for delivery [44]. Also once assembled into scaffolds, CNTs can be used as constructs for tissue engineering [45]. Recently, when CNT platforms were used to culture cardiomyocytes, the growth and electrical activity of cultured cardiomyocytes were enhanced. [46] The authors created multiwalled CNT platforms by depositing a solution of functionalized multiwalled carbon nanotubes onto glass coverslips and allowing the solution to dry. Neonatal rat ventricular myocytes were then seeded and observed for three days. The proliferation of rat cardiomyocytes on CNT platforms was enhanced more effectively compared to gelatin-coated substrates and CNT platforms with fibroblasts. The cell culture area of rat cardiomyocytes on CNT platforms was larger than on the gelatin coated scaffolds, which indicates a higher proliferative capacity of cardiomycytes on the CNT platforms. Additionally, the metabolic cycles and electrical activity (including action potentials) of rat cardiomyocytes on CNTs platforms were greatly improved relative to gelatin coated scaffolds [46]. These results show that nanotechnology can improve regenerative potential of the heart.

When heart tissue is damaged due to a heart attack, cardiac patches can be treated for site-specific regeneration. These patches are three dimensional porous biomaterial scaffolds seeded with healthy cardiomyocytes [28, 47, 48]. A major limitation of these scaffolds is their poor conductivity which inhibits cell-cell coupling and delays electrical signal propagation [49]. Dvit *et. al*. incorporated gold nanowires into alginate scaffolds which bridged the electrically resistant pore walls of alginate and improved electrical coupling between neighboring cadiomyocytes [50]. These nanowires are synthesized by anisotropic gold seed elongation and have average lengths of 1 µm and diameters of 30 nm. When electrically stimulated, the cells synchronously contracted and the tissues were thicker and better aligned than those grown on pristine alginate. The cells grown on these nanowired scaffolds also produced more proteins specific to muscle contraction and electrical coupling. This study shows that integration of conducting nanowires within three dimensional scaffolds increases the potential therapeutic value of current cardiac patches.

As previously stated, the effectiveness of nanoparticles has been investigated in inhibiting growth of lung carcinoma cells [36]. Nanoparticles have also been investigated in the treatment of damaged cardiac tissue. Dvir et al. have developed a nanoparticle that can target the infarcted heart [51]. This nanotechnology takes advantage of two consequences following a heart attack. Following myocardial infarction (MI), the blood vessels in the left ventricle become leaky [52]. This result resembles EPR and allows nanoparticle penetration. The second event utilized following MI is that there is an upregulation of angiotensin 1 (AT1) receptors [53], which can serve as a specific target identifying unhealthy cardiac tissue. The vehicle was a PEGylated liposome with approximately 145 nm in diameter. This liposome has the ability to carry a therapeutic payload that can be released in a controlled fashion. A peptide that recognizes the AT1 receptors was covalently attached to the carboxylic groups on the PEGylated liposome. This approach exemplifies a successful application of nanoparticle in in regenerative medicine based on an improved understanding of phenomena on the nanoscale.

## Perspective

The field of nanotechnology has witnessed an explosion in research efforts over the past decade. Specific advances have been seen in nanotechnology applications to biomedicine that have revolutionized the way we view disease diagnosis and treatment, and tissue regeneration and repair. Scientists and engineers working together within the framework of nanotechnology have worked diligently to not only develop and apply their devices and methods within their own laboratories but have also sought far-reaching interdisciplinary collaborations to identify novel applications for these technologies. Continuous advances in nanotechnology will undoubtedly be observed as science and technology progress. Maintaining an open dialogue between researchers, clinicians, and industry partners is crucial for translatability of these revolutionary technologies. Without question, it is a very exciting time to work in the field of nanotechnology toward regenerative medicine as the number of researchers expands; the findings and technologies advance exponentially; and new avenues for research and application are discovered. Altogether, nanotechnology has provided a new set of tools that can help researchers solve current problems involving both the pulmonary and cardiovascular systems. However, nanotechnology in regenerative medicine is still at an infant level and is a complicated interdisciplinary field which needs the collective collaboration of physicists, chemists, biologists, engineers, and clinicians. To help mature the exciting field of nanotechnology, researchers must unravel the mechanisms of cell-biomaterial interactions at the nanoscale and develop unique nanotechnology applications in regenerative medicine.
